# Spider dynamics under vertical vibration and its implications for biological vibration sensing

**DOI:** 10.1098/rsif.2023.0365

**Published:** 2023-09-13

**Authors:** Jun Wu, Thomas E. Miller, Alice Cicirello, Beth Mortimer

**Affiliations:** ^1^ Department of Biology, University of Oxford, Oxford, UK; ^2^ Department of Engineering Science, University of Oxford, Oxford, UK; ^3^ Department of Engineering Structures, Section of Mechanics and Physics of Structures, Delft University of Technology, Delft, The Netherlands

**Keywords:** modelling, spiders, modal test, dynamics, vibration sensing

## Abstract

Often overlooked, vibration transmission through the entire body of an animal is an important factor in understanding vibration sensing in animals. To investigate the role of dynamic properties and vibration transmission through the body, we used a modal test and lumped parameter modelling for a spider. The modal test used laser vibrometry data on a tarantula, and revealed five modes of the spider in the frequency range of 20–200 Hz. Our developed and calibrated model took into account the bounce, pitch and roll of the spider body and bounce of all the eight legs. We then performed a parametric study using this calibrated model, varying factors such as mass, inertia, leg stiffness, damping, angle and span to study what effect they had on vibration transmission. The results support that some biomechanical parameters can act as physical constraints on vibration sensing. But also, that the spider may actively control some biomechanical parameters to change the signal intensity it can sense. Furthermore, our analysis shows that the parameter changes in front and back legs have a greater influence on whole system dynamics, so may be of particular importance for active control mechanisms to facilitate biological sensing functions.

## 1. Introduction

In engineering, vibration sensing is an important technology for localizing sources of interest and monitoring or detecting potential issues with equipment, which can avoid accidents or economical loss [1,2]. In biology, vast numbers of animals are adept at vibration sensing, which for arthropods is usually performed through sensors embedded in their numerous legs [3,4]. Among them, spiders are arguably the vibration sensing experts in the animal kingdom, which enables them to detect prey, find mates and avoid predators effectively [5,6]. Understanding how spiders sense vibrations is helpful for developing innovative bioinspired technologies for use in engineering [7–9].

**Table 1.** Nomenclature.

symbols	
Im(∗), Re(∗)	imaginary part and real part of *, respectively
$I_{x}$, $I_{y}$	moments of inertia of the spider body in pitch (*x*) and roll (*y*) directions, respectively
j	imaginary unit $j=\;\sqrt{-1}$
$k_{ijn}$, $c_{ijn}$	stiffness and damping coefficient of the connection between leg *ijn* (see §3.1) and the body
$k_{ijn}^{g}$, $c_{ijn}^{g}$	stiffness and damping coefficient of the contact between leg *ijn* (see §3.1) and the ground
m	total mass of the spider
$m_{b}$	mass of the spider body, including abdomen and cephalothorax
$m_{ijn}$	mass of leg *ijn* (see §3.1)
t	time
V	wave speed
$w_{b}$	bounce of the spider body in the model
β	decay rate
$\varphi_{b}$, $\theta_{b}$	pitch and roll of the spider body in the model, respectively
λ	wavelength
abbreviations	
DOF	degree of freedom
EoM	equation of motion
FRF	frequency response function
MAC	modal assurance criterion
WMAC	weighted modal assurance criterion

The vibration receptors of spiders are highly sensitive, capable of sensing external vibrational stimuli with amplitudes down to tens of nanometres [10,11]. Since spiders are found in diverse environments but have similar basic body plans, spiders are therefore good model organisms for studying vibration sensing [8,12]. The organs involved in mechanosensation, whether vibration sensing from external or internal sources, include slit sensilla, which act as detectors of strain of the spider's exoskeleton [13]. The slits, often parallel to the leg axis, have a membrane across them and mechanosensory cells attached beneath that send a nerve impulse when the slit is deformed perpendicular to the axis of slit [13,14]. Slit sensilla are often co-located to form groups of slits and lyriform organs [15,16]. Some are more sensitive to external vibrational stimuli than other slit sensilla. The metatarsal lyriform organs, located on the distal end of the spider metatarsus (towards the claw end of the spider leg), show high vibration sensitivity [11,17]. Slits on the pretarsal claw are also vibration sensitive [18]. However, other lyriform organs, generally situated close to leg joints, detect internally generated vibrations, for example being involved in kinaesthetic orientation [19].

To be detected by the spider, external vibration stimuli have two mediums through which the vibration propagates before reaching the mechanosensors: the material in the environment (e.g. spider webs) and the spider body [8]. For spiders that build webs, the webs play an important role in shaping vibrational information [20], whether for detection and localization of prey, objects or potential mates. The vibration transmission through different kinds of webs has been investigated in orb webs [20,21], sheet and tangle webs [22]. Other spiders detect vibrations along other substrates such as the ground or mixed media [23,24]. Regardless of the substrate, the spider is able to discriminate between vibration sources and orientate/locate these sources [21].

Compared with effects of the substrate, particularly webs, the dynamics of the spider itself has been relatively overlooked. In fact, experimental studies of web vibration have often not included the spider, yet inertial effects of the added mass of the spider are important for vibrational motion of the whole system [25]. By dynamics of the spider, we mean not only the motion of the spider legs, cephalothorax and abdomen combined, but also the interaction with the multiple points of contact with the vibrating surface that the spider is standing on. Previous studies have been focused on individual organs and leg segments in isolation [11,15,26]. To our knowledge, little research related to whole-body dynamics of spiders has been published to date. The dynamic properties of the spider, including the natural frequencies, mode shapes and damping ratios, can have a significant influence on vibration transmission [8]. We would also expect the influence of spider dynamics to change with substrate, as webs may in turn be influenced by the motion of the spider on it, whereas this is less likely for more dense substrates.

One key biological function of vibration sensing is to determine the source location, which spiders do through comparison of vibrational inputs from lyriform organs on all eight legs [27,28]. This has been evidenced through ablation of the tibial and femoral lyriform organs, which reduces the directional accuracy of orientation [29]. Ground-dwelling spiders show a preference for certain substrate types to maximize transmission efficacy for communication [23,24]. The chosen hunting substrates of spiders such as *Cupiennius salei*, which are sit-and-wait predators on the surface of large, broad-leaved, mechanically strong plants such as bromeliads, also possibly implicates a role for substrate preference in a hunting context [10]. Web-building spiders possess even more control over the vibration transmission properties. Web-building spiders possess far more control over the vibration transmission properties of their substrate as they engineer it themselves, which means the degree of physical constraints may differ between orb weavers and terrestrially hunting spiders [30].

Source localization in spiders is dependent on the spider body–substrate combined vibrational dynamics. This means that variation in the biomechanical parameters of the spider body would be expected to affect the ability of the spider to accurately localize vibrational sources. Some of these parameters can theoretically be controlled by the spider through changes in leg mechanical properties via mechanisms such as hydrostatic pressure [31] or muscle action [32] that vary leg joint stiffness and damping, and even changes in posture that vary leg span and angle [33]. Other parameters will be dependent on physiological state or other constraints such as body mass, inertial or mass distribution, and body size and scaling that will vary with developmental stage and nutritional state [34–36]. In the natural system, variation in a single biological trait can influence several of these biomechanical parameters at once, which can make it difficult to study their effects experimentally. Using a modelling approach we can examine the effects of biomechanical parameters in isolation, which can give insights into the physical constraints acting on vibration source localization.

To this end, the motivation of this paper is to study the dynamics of a freely vibrating, untethered live spider in its natural posture using laser vibrometry in order to develop models of its vibration dynamics. We use the tarantula *Grammostola pulchra*, a terrestrial hunter which is ideally suited for vibrometry experiments (it retains a constant posture for extended periods). This species hunts on soily substrates of grasslands [37]. With these data, we perform a modal test and develop a corresponding mathematical model to describe the general biodynamics of the spider on a vibrating surface. Using the model, we study how changes in mechanical parameters (table 1) including mass/inertia, stiffness, damping and leg length/angle influence its vibration transmission, including the interaction with vibration source location and substrate wave speed, and hence the overall effect on vibration source localization. Knowing how different parameters influence the vibration transmission through the spider body provides cues for how the spider may control its body to aid in vibration sensing or be limited by physical parameters outside of their control.

## 2. Experimental set-up

**Figure 1.**
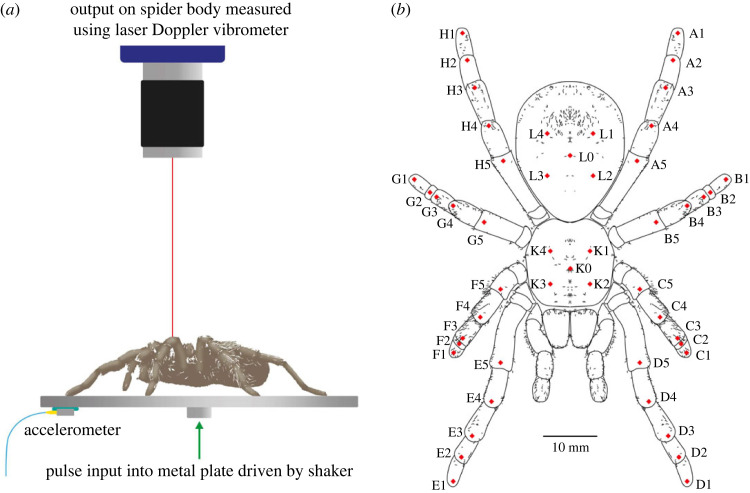
Experiment: (*a*) set-up; (*b*) measurement points on the spider. This figure is used for illustration of all the measurement points, given by the red dots in (*b*). Note the posture shown here is not the same as the test. During experiments, spiders were not anaesthetized or tethered but a tube was used to enclose the spider.

Modal test is a commonly used experimental method to derive the modal properties of a dynamic or biodynamic system, i.e. the inherent frequency-specific vibration characteristics [38,39]. The modal properties of the spider (i.e. substrate independent) were investigated using the set-up shown in figure 1 and electronic supplementary material, figure S1. We used immature specimens of *Grammostola* *pulchra* from our captive laboratory population. The spiders were kept in plastic terrariums with damp coir substrate and fed with crickets twice weekly, with water available in a dish. The animals were housed in a temperature-controlled room set at 20°C, on a 12/2 h day/night cycle. An untethered live spider (tarantula *Grammostola pulchra*) in its natural posture was put on an aluminium circular plate. The plate was connected to the top of a shaker (Modal Shop K2004E01) using a threaded bolt. The out-of-plane acceleration of the plate was measured by an accelerometer (PCB 352A21). The vertical velocity response of the spider was measured by a laser Doppler vibrometer (LDV) (Polytec VibroOne), whose advantage is it enables non-contact measurement as opposed to other measurement methods, e.g. strain gauge and accelerometer. The distance between the vibrometer and the spider was adjusted to maximize the signal-to-noise ratio. A circular tube with no contact with the plate was used to enclose the plate during the test to avoid the spider escaping.

During the test, a pulse with a duration of 1.28 s and pulse width of 0.2 ms was used as the excitation and was generated by the shaker. Before the formal test, the response on the plate without the spider was measured under the pulse excitation, which was to ensure no resonances of the plate in the frequency range up to 500 Hz would affect the response. During the formal test, the spider had a constant posture (where any movement detected by visual inspection would terminate the test). The modal test followed the single-input multiple-output (SIMO) method [40]. The pulse excitation was repeated while each time the responses on the spider were measured by focusing the laser beam at one of the 50 different locations, including 5 points per leg, 5 points on the abdomen and 5 points on the cephalothorax, as shown in figure 1*b*. At each point, the measurement was repeated three times. Each point on the leg is named using a letter (standing for the leg) followed by a number (standing for its position on the leg). The legs are named using the letters A–H from the left posterior leg to the right posterior leg in the clockwise direction, while the 5 points on each leg are named using the numbers 1–5 from the distal joints to the proximal joints. Each point on the abdomen and cephaolthorax is named using the letter L and K respectively, followed by a number 0–4 for its position. All the data were acquired by the Polytec system with a sampling frequency of 25 600 Hz. The frequency response functions (FRFs), i.e. the ratio between the response (output) and excitation (input) expressed as a function of the excitation frequency, were calculated from the acceleration measured on the plate to the accelerations on the spider that are transformed from the velocity measured by the LDV. The FRFs are used to quantify the responses of the spider to the excitation. In this paper, the FRFs are calculated between two vibration quantities with the same unit, so they are also transmissibilities.

## 3. Modelling and analysis methods

### 3.1. Model description

**Figure 2.**
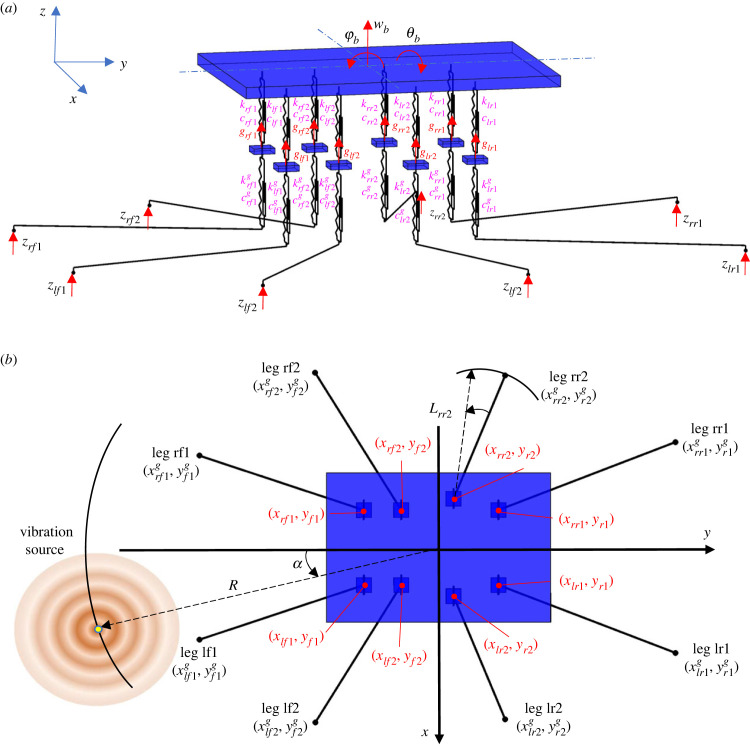
The lumped parameter model of a spider: (*a*) schematic diagram; (*b*) top view. The black horizontal lines are massless and artificial, only for distinguishing the leg input locations from leg–body connection locations. All parameters are explained in §3.1.

Generally speaking, the modelling of the spider is very similar to the full car model [41,42] in vehicle engineering, meaning that approaches from engineering can be applied to this novel context of exploring the biodynamics of the spider. The low model complexity brings about several advantages, including (1) it enables us to focus on the most critical aspects of the spider dynamics and simplify the representation while being sufficient to fulfil our objectives; (2) it is computationally less demanding; (3) it is more interpretable and easier to understand; (4) it is more robust and less prone to overfitting. Here we describe the components and parameters of the model, including relative locations, where the details of how the parameters are determined are given in electronic supplementary material, S2. As shown in figure 2, the model includes the spider body and eight legs with a total of 11 degrees of freedom (DOF). To characterize the global vertical dynamics of the spider and focus on the most critical aspects of the spider dynamics while simplifying the representation, only the vertical vibration and the rigid-body motion of the spider body and legs are considered. The spider body (including abdomen and cephalothorax) is modelled as a rigid body with 3 DOF, i.e. bounce $w_{b}$, pitch $\varphi_{b}$ and roll $\theta_{b}$. Each of the 8 legs of the spider is modelled as a rigid body with bounce DOF $g_{ijn}$ (i=l,r; j=f,r; n=1,2). The subscript denotes the leg name, in which the first letter *l* or *r* stands for the left or right leg, and the second letter *f* or *r* stands for the anterior or posterior leg, and the final number 1 or 2 for the first or second leg (figure 2*b*). Each leg has a mass of $m_{ijn}$. The coordinates of the locations where each leg is in connection with the spider body are defined as $\left(x_{ijn},y_{\;jn}\right)$, and where the claw of each leg is in contact with the ground are defined as $\left(x_{ijn}^{g},y_{\;jn}^{g}\right)$. Due to the symmetry about the mid-sagittal plane, it is satisfied that $m_{ljn}=m_{rjn}$, and $x_{ljn}=-x_{rjn}$, $x_{ljn}^{g}=-x_{rjn}^{g}$ as left and right sides are identical. The connection between the spider body (modelled here as combined cephalothorax and abdomen) and each leg is modelled as Kelvin–Voigt model by a spring–damper element with stiffness of $k_{ijn}$ and damping coefficient of $c_{ijn}$ [43]. The contact between each leg and the ground is also modelled as Kelvin–Voigt model by a spring–damper element with stiffness of $k_{ijn}^{g}$ and damping coefficient of $c_{ijn}^{g}$. There is a vertical excitation $z_{ijn}$ for leg *ijn*, whose coordinate is $\left(x_{ijn}^{g},y_{\;jn}^{g}\right)$.

In terms of model inputs, the model uses either an equal input into each leg corresponding to the modal test, or a point vibration source excitation for parametric study. In the latter case, the source location is defined by the distance from the centre of mass (the origin) of the spider (R) and an angle α (positive if anti-clockwise) with respect to −*y* axis, as shown in figure 2*b*.

### 3.2. Equations of motion and model calibration

The equations of motion (EoM) of the spider model are derived according to Newton's second law (electronic supplementary material, S1.1). Based on the EoM, modal analysis is carried out (electronic supplementary material, S1.2), from which the modal frequencies, damping ratios and mode shapes of the model can be obtained. The FRFs are calculated (electronic supplementary material, S1.3) based on the model under equal inputs for all eight legs corresponding to the modal test or under a point vibration source excitation for parametric study.

To make the model have a good consistency with the experiment, model calibration is carried out considering four objectives (electronic supplementary material, S2). Objective 1 is to minimize the difference of the natural frequencies between the model and experiment. Objective 2 is to minimize the difference of the damping ratios between the model and experiment. Objective 3 is to maximize the resemblance of the mode shapes between the model and experiment, while objective 4 is to minimize the difference of the FRFs between the model and experiment under equal leg inputs. The natural frequencies, damping ratios and mode shapes (considered in the first three objectives) are the natural properties of the spider, independent of excitation. They are the same under equal input and under point vibration source input, so the model calibrated using the equal input can be applicable to the following parametric studies under point vibration source input. The fourth objective is complementary and optional, which is used to ensure the vibration transmission under equal input is accurate. The four objectives can be balanced by setting suitable weighting factors in electronic supplementary material, equation (S18). The obtained parameters are listed in electronic supplementary material, table S1. Then sensitivity analysis is carried out to determine the confidence we have in the accuracy of the parameters (electronic supplementary material, S3).

## 4. Results and discussion

Our aim in this paper was to investigate the dynamics of an untethered spider on a vibrating surface and its implications for spider vibration sensing. Using a modal test based on laser vibrometry data, we reveal the modal properties of the spider and then develop and validate a mathematical model of the spider to simulate its dynamics (§4.1). We then use a parametric study approach on the calibrated model to investigate the influence of different selected parameters on the dynamic response of the spider–vibrating surface system (§4.2). This provides insights into the relative influence of different mechanical parameters on spider dynamics. Furthermore, it reveals which parameters act as physical constraints or which can be actively controlled by the spider to influence vibration sensing, with a focus on the influence of parameters on source localization.

### 4.1. Modal properties and model validation

The modal identification was performed using the PolyMAX method [44], and the results are shown in electronic supplementary material, S4. The frequency above 200 Hz is not considered because the FRFs above 200 Hz have low amplitude and no obvious resonance frequencies while the pulse excitation can theoretically excite up to 5 kHz. In the modal test, five modes are identified in the frequency range of 20–200 Hz, as shown in table 2. The first two modes are dominated by combined bounce and pitch motions. The mode shape of the first mode features the in-phase upward bounce and forward pitch (electronic supplementary material, video S1) while the mode shape of the second features the out-of-phase upward bounce and forward pitch (electronic supplementary material, video S2). The third mode is a whole-body upward bounce mode in phase with slight forward pitch of the cephalothorax (electronic supplementary material, video S3). The fourth mode shape is very similar to the third, which features whole-body upward bounce out of phase with slight forward pitch of the cephalothorax (electronic supplementary material, video S4). The fifth mode is dominated by the bounce of cephalothorax and all the legs with minor vibration of the abdomen (electronic supplementary material, video S5).

**Table 2.** The comparison of the modal properties between the model and experiment. The coordinates of the points in the experimental mode shapes are measured from the spider under test. **‘**WMAC’ (weighted modal assurance criterion) only applies to mode shape, and **‘**error’ only applies to frequency and damping ratio. See figure 1*b* for a full description of experimental laser vibrometry locations on the spider. See also electronic supplementary material, videos S1–S7, that show mode shapes.

mode	property	experiment	model	WMAC	error
1	mode shape	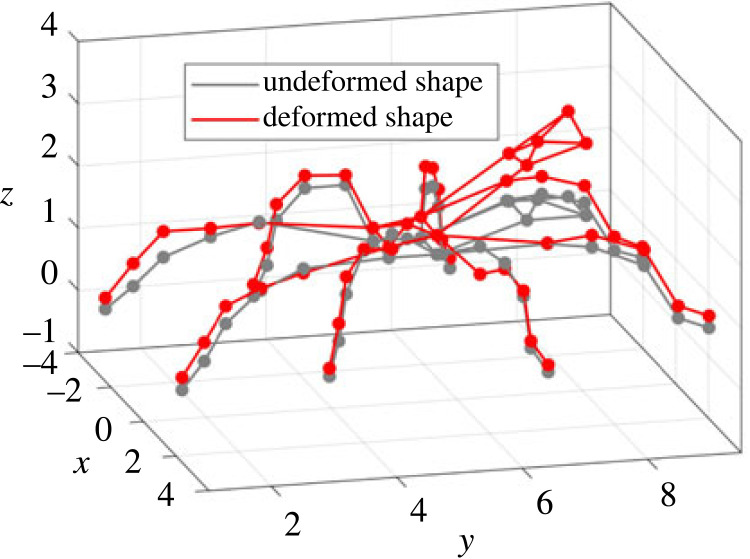	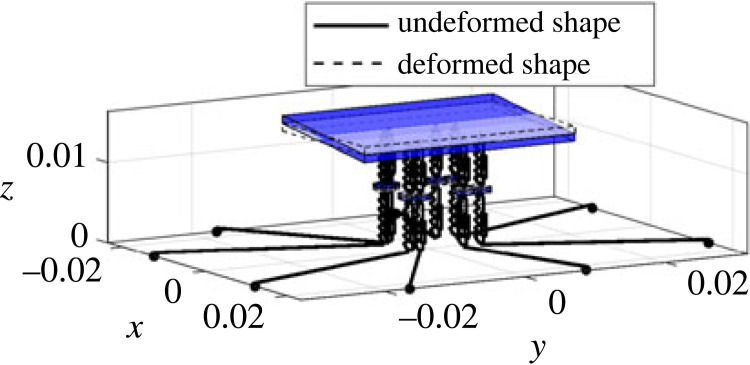	<0.01	—
	description	in-phase upward bounce and forward pitch	—	—	—
	frequency (Hz)	31.75	31.83	—	<1%
	damping ratio	0.227	0.228	—	<1%
2	mode shape	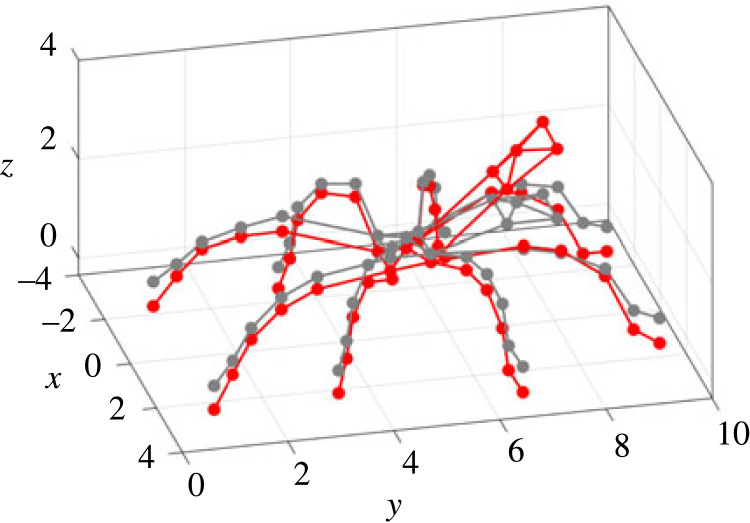	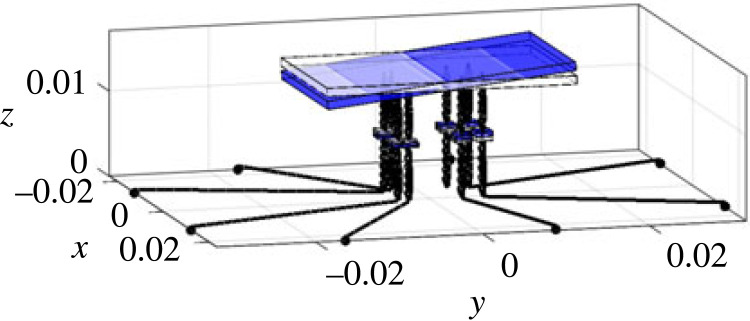	0.59	—
	description	out-of-phase upward bounce and forward pitch	pitch of the spider body	—	—
	frequency (Hz)	37.65	36.79	—	2.3%
	damping ratio	0.175	0.070	—	60%
3	mode shape	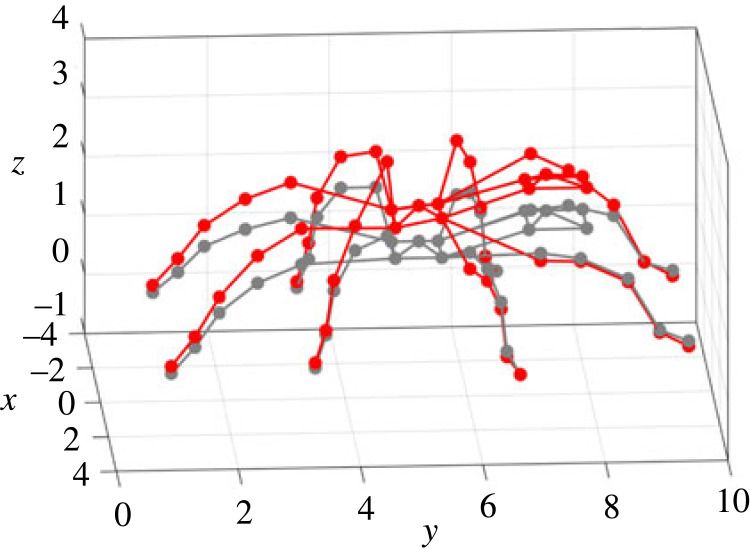	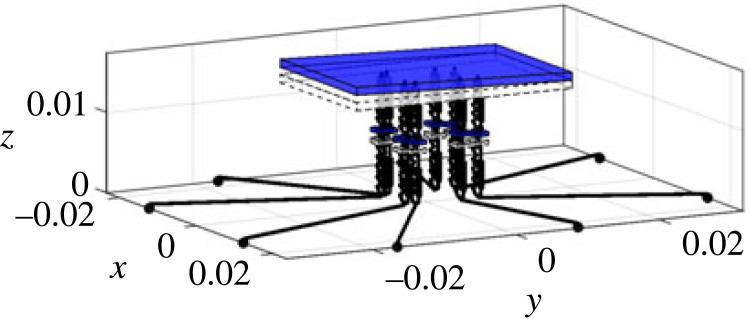	0.83	—
	description	whole-body upward bounce in phase with slight forward cephalothorax pitch	bounce of the spider body and all legs with slight body pitch	—	—
	frequency (Hz)	74.15	70.12	—	5.4%
	damping ratio	0.092	0.229	—	148.9%
4	mode shape	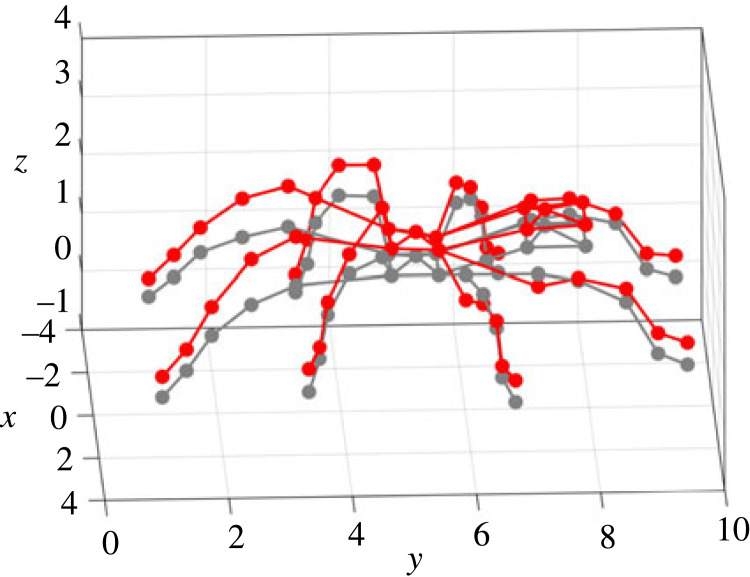	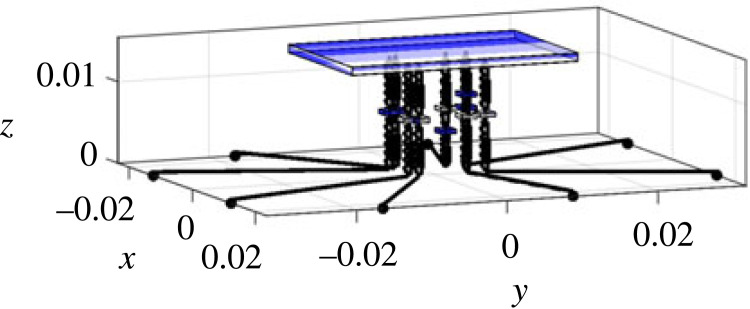	0.016	—
	description	whole-body upward bounce out of phase with slight forward cephalothorax pitch	—	—	—
	frequency (Hz)	108.43	109.51	—	<1%
	damping ratio	0.076	0.090	—	18.4%
5	mode shape	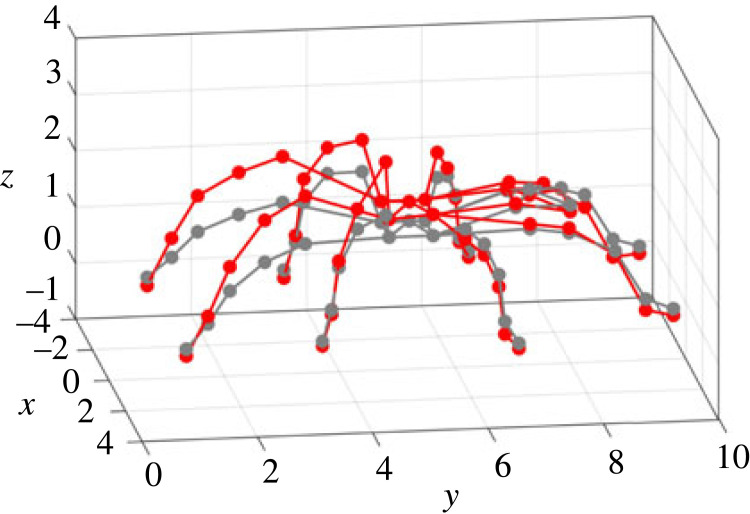	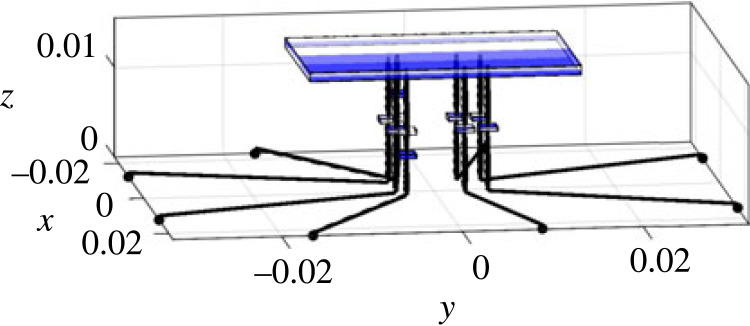	<0.01	—
	description	bounce of cephalothorax and all the legs with minor abdomen vibration	—	—	—
	frequency (Hz)	137.41	137.17	—	<1%
	damping ratio	0.103	0.116	—	12.6%

On the one hand, it is well known that the signal energy generated by insects is dominated by frequencies in the range less than 1000 Hz [6]. The resonances of the spider body lie within this range (below 200 Hz; note theoretical spider–web coupled systems are also in this range less than 20 Hz [45]), opening the possibility that changing body dynamics can enable higher amplitude sensing of the vibration signal. On the other hand, the spider dynamics makes it act like a low-pass filter when transmitting vibration to the slit sensilla. To sense all vibration frequencies equally below 1000 Hz, the slit sensilla need to be more sensitive to higher frequencies than lower frequencies, which is consistent with the tuning curves of slit sensilla [6].

The model parameters after calibration (which include masses, moments of inertia, stiffness, damping and coordinates of connection between legs and body) and the weighting factors are listed in electronic supplementary material, table S1. The frequencies of the model are in good agreement with the experiment with all errors less than 6% (table 2). The damping ratios of 1st, 4th and 5th modes in the model are relatively consistent with the experiment (error less than 19%), while the damping ratios for the other two modes (2nd and 3rd) are not satisfactory (error greater than 60%). The 2nd and 3rd mode shapes of the model are generally in agreement with the experiment, while the other mode shapes are not. The 2nd mode shape in the model is dominated by the pitch of the spider body (electronic supplementary material, video S6), while the 3rd mode shape is dominated by the bounce of the spider body and all legs with slight body pitch (electronic supplementary material, video S7). It should be noted that the 2nd and 3rd modes should be more important than the others under equal leg inputs since their peaks are more prominent in the experimental FRFs (electronic supplementary material, figures S2 and S3). Overall, the mode shapes of the spider tend to combine bounce motions of the whole body with pitch of the cephalothorax and abdomen, either in or out of phase. It should be noted that the FRFs in electronic supplementary material, figures S2 and S3, are not perfectly symmetrical, and the potential reasons for bilateral asymmetry include: (1) the cumulative effect of small differences in leg joint angles between the left and right sides of the spider's body (electronic supplementary material, figure S1); (2) larger variation in biomechanical parameters, such as muscle contraction and haemolymph pressure differences between left and right sides of the body. However, for simplicity and reducing model parameters, bilateral symmetry is assumed in the modelling.

**Figure 3.**
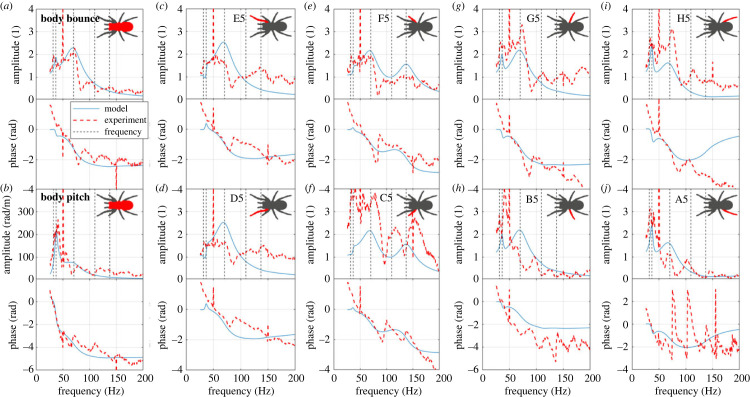
The comparison of the amplitudes and phases of FRFs between the model and experiment for (*a*) body bounce; (*b*) body pitch; (*c*) E5; (*d*) D5; (*e*) F5; (*f*) C5; (*g*) G5; (*h*) B5; (*i*) H5; (*j*) A5. Vertical dashed lines represent the natural frequencies of the model.

The comparison of the FRFs (including the amplitude and phase) between the model and experiment is shown in figure 3. The FRFs of the model show satisfactory agreement with the experimental FRFs, although the experimental FRFs are quite noisy, especially for the body bounce, body pitch, A5, F5, G5 and H5. This means, despite its simplicity and relatively few parameters and DOFs, the model can be adequately used to quantify the dynamics and vibration transmission of the spider.

In terms of the limitations of the model, due to the simplicity of the model the mode shapes of the spider cannot be reproduced perfectly, especially for the modes with complicated bending of the legs, or with different motions for the abdomen and cephalothorax. Furthermore, only vertical vibration is considered here under transverse wave excitation. However, in the natural context, substrate vibrations propagate not only as transverse waves, but also as other wave types such as longitudinal waves [46]. In terms of the limitation of the modal test, with our approach using an equal vertical excitation into each leg, the roll vibration may not be excited due to the approximate symmetric property of the spider about the mid-sagittal plane. Thus, it cannot be ruled out that there may be modes dominated by the roll motion of the spider body in the natural context in the considered frequency range. For example, in the real system, it is possible that roll plays a more significant role when inputs into all eight legs are asymmetrical or when asymmetrical postures are adopted by the spider. Future modal analyses should consider more of the three-dimensional motions of the spider system under different excitation regimes.

### 4.2. Vibration transmission

To investigate the implications of spider dynamics on spider vibration sensing, we moved to a more realistic environment where there is a point vibration source which propagates through a substrate and the coupled spider system. Using our calibrated mathematical model of the spider, this allows us to start to investigate the inter-leg differences between the legs in vibration transmission (§4.2.1). We then used the model to conduct a parametric study when there is a point vibration source, where parameters are varied systematically to determine their effect on spider dynamics, especially differences between the legs. The vibration source is assumed to be in a sinusoidal form. The parameters for the vibration source are listed in table 3, in which the speed *V* and decay rate β of the vibration source are obtained from [47] on wet sand, which is within the right order of magnitude for wet soily substrate of tarantulas [37].

**Table 3.** The parameters of the vibration source. The superscript 1 means the parameters were obtained from the literature [47] and the superscript 2 means arbitrarily chosen for a point vibration source scenario.

parameters	values	parameters	values
$V^{1}$	80 m s^−1^	$R^{2}$	20 cm
$\beta^{{\;}^{1}}$	6.977 m^−1^	$\alpha^{2}$	10°

The parameters we investigated include factors relating to the spider, including mass and inertia (§4.2.2), leg stiffness and damping (§4.2.3) and leg span and angle (§4.2.4), but also factors relating to the environment, such as wave speed (§4.2.5). All the parameters are kept the same as the reference parameters in electronic supplementary material, table S1, except the varying ones. We are focusing on the influence of parameter changes on the vibration transmissions to the spider body and all legs, which are quantified by FRFs.

#### 4.2.1. Inter-leg difference

**Figure 4.**
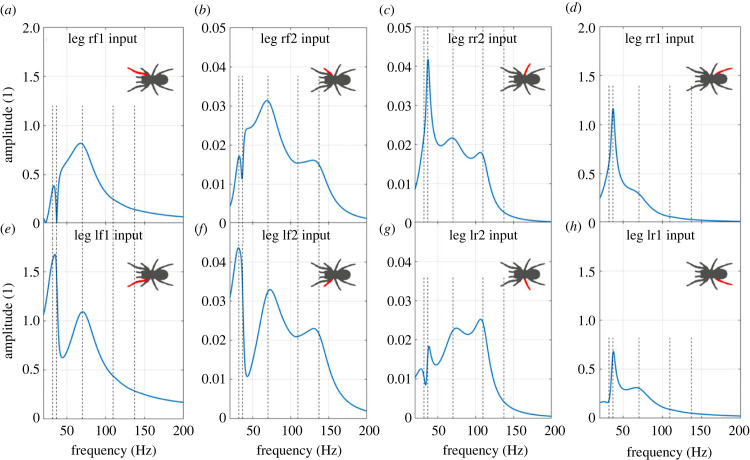
The FRFs to the bounce of leg lf1 from (*a*) leg rf1 single leg input, (*b*) leg rf2, (*c*) leg rr2, (*d*) leg rr1, (*e*) leg lf1, (*f*) leg lf2, (*g*) leg lr2, (*h*) leg lr1 single leg input. Note the different axes for (*b*), (*c*), (*f*) and (*g*). The vertical dashed lines represent the natural frequencies of the original system.

We start with how the vibration transmissions from different leg inputs differ when there is only one leg input at a time (electronic supplementary material, equation (S7)). This simplified vibration input scenario allows us to investigate the relative contribution of different legs to spider vibration transmission. Figure 4 gives the FRFs from different single leg inputs to the bounce of leg lf1 (the output). It shows how the FRFs from different leg inputs differ, due to the difference in the vibration transmission through different legs.

This shows that inputs from the front and back leg inputs are easily transmitted to the other legs, while the transmissions from the middle four leg inputs are quite low except the within-leg transmission. This inter-leg difference is independent of the vibration source as it is a function of the model properties. Since the response of each leg or body is the summation of the vibration transmissions from all eight leg inputs, it can be expected that the parameter changes in the front and back legs have a greater influence on other legs, while the changes in each of the middle four legs may only affect that specific leg. This can generally be applied to the parameters investigated in the subsequent sections. The spider leg responses are therefore more robust to changes in biomechanical parameters in the middle four legs, while variation in the parameters in the front and back four legs will have a much greater influence on the spider leg responses.

#### 4.2.2. Influence of body mass and inertia

**Figure 5.**
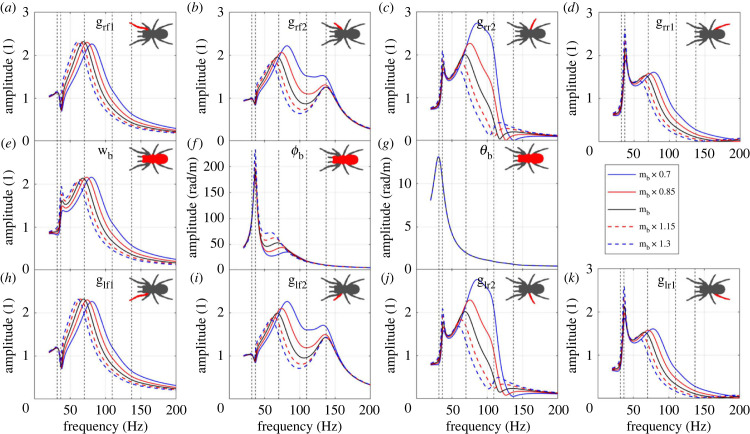
The influence of body mass $m_{b}$ on the FRFs to (*a*) leg rf1 bounce; (*b*) leg rf2 bounce; (*c*) leg rr2 bounce; (*d*) leg rr1 bounce; (*e*) body bounce; (*f*) body pitch; (*g*) body roll; (*h*) leg lf1 bounce; (*i*) leg lf2 bounce; (*j*) leg lr2 bounce; (*k*) leg lr1 bounce. The vertical dashed lines represent the natural frequencies of the original system.

The influence of the body mass on the FRFs is plotted in figure 5. The FRFs in all subsequent subsections are calculated from leg lf1 input to all DOFs according to electronic supplementary material, equation (S11). The FRFs show significant decrease of the third resonance frequency and its amplitude with the increasing body mass, which is because the increase of the body mass can increase the modal mass of the third mode that is dominated by the body bounce (table 2), and the increase of the modal mass reduces the resonance frequency and the FRF amplitude according to single DOF system [48]. The effect is not equal between all four legs, with variation in mass having a greater influence on the middle four legs (lr2 and rr2 in particular).

The change of the body mass leads to the change of the moment of inertia. The influence of the moment of inertia in pitch direction $I_{x}$ on the FRFs is plotted in electronic supplementary material, figure S4. The FRFs show significant decrease of the second resonance frequency and its amplitude with the increasing moment of inertia. This is because the increase of moment of inertia increases the modal mass of the second mode that is dominated by the body pitch (table 2).

Overall, changes in mass, and linked moment of inertia, have effects on the frequency and amplitude of two major spider vibration modes. Short-term mass variation is influenced by several biological factors, including feeding status, hydration and reproduction, which may have some impact on the spider dynamics and so vibration sensing in the frequency range under 200 Hz.

#### 4.2.3. Influence of stiffness and damping

**Figure 6.**
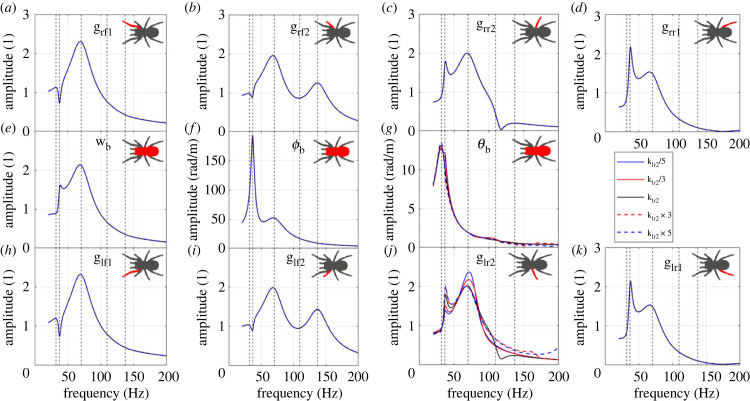
The influence of $k_{lr2}$ on the FRFs to (*a*) leg rf1 bounce; (*b*) leg rf2 bounce; (*c*) leg rr2 bounce; (*d*) leg rr1 bounce; (*e*) body bounce; (*f*) body pitch; (*g*) body roll; (*h*) leg lf1 bounce; (*i*) leg lf2 bounce; (*j*) leg lr2 bounce; (*k*) leg lr1 bounce. The vertical dashed lines represent the natural frequencies of the original system.

To illustrate how the leg stiffness can influence the vibration transmission, as an example, the influence of the leg–body connection stiffness of the leg lr2 ($k_{lr2}$) is plotted in figure 6. The vibration transmission to leg lr2 is influenced by changing its stiffness, while the vibration transmissions to other legs remain the same. This is consistent with our point in §4.2.1 (figure 4) that the parameter changes in one of the middle four legs only influences itself. It can be expected that if stiffness of the front or back legs is changed, the vibration transmissions to all the legs will be changed. To be specific, the amplitude of the second resonance (corresponding to mode 2) is increased while the amplitude of the third one (corresponding to mode 3) is reduced with the increasing stiffness. Only slight shift of the resonance frequencies is observed with changing leg stiffness.

**Figure 7.**
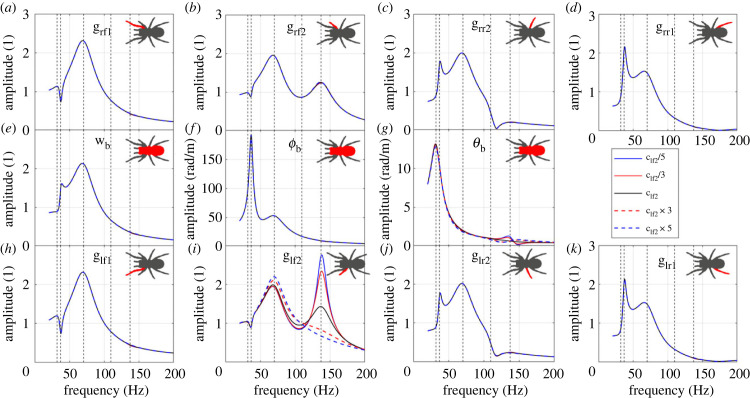
The influence of $c_{lf2}$ on the FRFs to (*a*) leg rf1 bounce; (*b*) leg rf2 bounce; (*c*) leg rr2 bounce; (*d*) leg rr1 bounce; (*e*) body bounce; (*f*) body pitch; (*g*) body roll; (*h*) leg lf1 bounce; (*i*) leg lf2 bounce; (*j*) leg lr2 bounce; (*k*) leg lr1 bounce. The vertical dashed lines represent the natural frequencies of the original system.

To illustrate how the leg damping can influence the vibration transmission, as an example, the influence of the leg–body connection damping of the leg lf2 ($c_{lf2}$) is plotted in figure 7. The vibration transmission to leg lf2 is changed when its leg damping is changed, while the vibration transmissions to other legs remain the same (also consistent with §4.2.1). To be specific, the amplitude of the third resonance (corresponding to mode 3) is increased, while the amplitude of the fifth resonance (corresponding to mode 5) is suppressed significantly with the increasing damping coefficient. Only a slight shift of the resonance frequencies is observed with changing leg damping.

Leg stiffness and damping are influenced by several biological factors including hardening of the cuticle after moulting [49], leg angle, as well as active muscle action [32] and hydrostatic pressure [31]. They can also be affected by the viscoelastic cuticle material structure, where specific temperature/humidity/strain rate (i.e. excitation frequency) combinations can alter material structure–property links via glass transition effects [50,51]. These parameters could be seen as a constraint set by the mechanical properties of the spider, but equally the spider has some degree of control of these—for instance, increasing joint angle stretches the articular membrane, increasing alignment in the material and hence stiffness [32]. Consistent with our findings here, leg joint viscoelasticity is dependent on the vibration direction, which may be used by the spider to control the signal amplitude transmitted to the mechanoreceptors [32]. Therefore, it is possible that leg stiffness and damping could be used by the spider to actively control its vibration sensing, which will require further study to investigate.

#### 4.2.4. Influence of leg span and angle

The vibrational input that goes into the spider is influenced by the leg–ground contact locations. In turn these are influenced firstly by leg span and the leg angle, as shown in figure 2*b*, but also properties of the substrate such as wave speed (§4.2.5).

**Figure 8.**
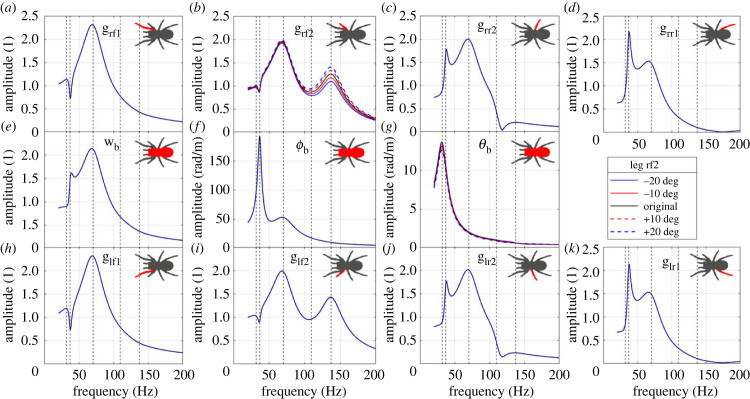
The influence of the angle of leg rf2 on the FRFs to (*a*) leg rf1 bounce; (*b*) leg rf2 bounce; (*c*) leg rr2 bounce; (*d*) leg rr1 bounce; (*e*) body bounce; (*f*) body pitch; (*g*) body roll; (*h*) leg lf1 bounce; (*i*) leg lf2 bounce; (*j*) leg lr2 bounce; (*k*) leg lr1 bounce. The vertical dashed lines represent the natural frequencies of the original system. The change of leg angle is positive if anti-clockwise (figure 2*b*).

**Figure 9.**
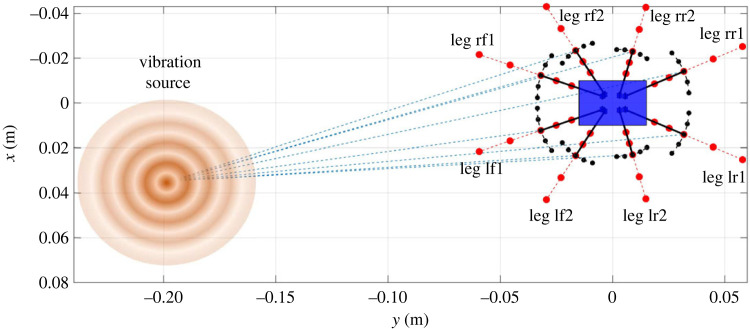
Schematic diagram showing the change of leg angle and span. The black dots stand for the 5 positions in the case of changing the leg angle, while the red dots for the 5 positions in the case of changing the leg span.

The influence of the angle of a single leg (rf2) is shown in figure 8. Changing this angle has a significant influence on the FRF to leg rf2, while has no obvious influence on the FRFs to other legs (§4.2.1). In this case, a positive/anti-clockwise change of leg angle increases the FRF amplitude to leg rf2 above 100 Hz, dominated by the fifth mode. This is because the change of the angle of leg rf2 is almost along the line connecting the vibration source and leg–ground contact position (figure 9). In addition, there is no obvious shift of the resonance frequencies, which is because the change of the leg–ground contact position does not affect the natural frequencies of the spider.

**Figure 10.**
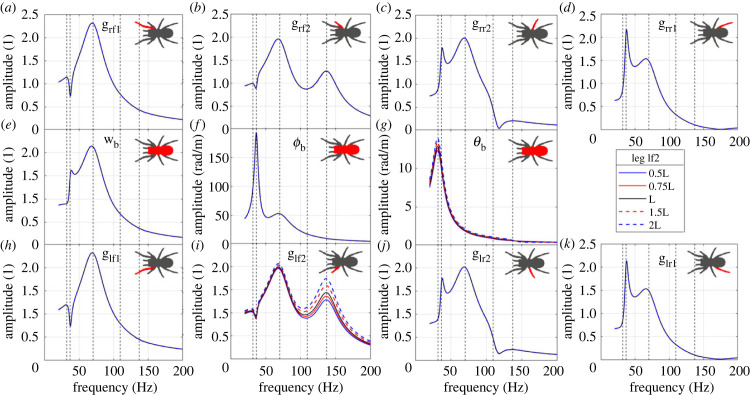
The influence of leg lf2 span on the FRFs to (*a*) leg rf1 bounce; (*b*) leg rf2 bounce; (*c*) leg rr2 bounce; (*d*) leg rr1 bounce; (*e*) body bounce; (*f*) body pitch; (*g*) body roll; (*h*) leg lf1 bounce; (*i*) leg lf2 bounce; (*j*) leg lr2 bounce; (*k*) leg lr1 bounce. The vertical dashed lines represent the natural frequencies of the original system.

The influence of leg lf2 span is shown in figure 10. The change of the span has a significant influence on the FRF to leg lf2, while it has no obvious influence on the FRFs to other legs. In this case, an extension of the leg forward increases the FRF amplitude to leg lf2 above 100 Hz, dominated by the fifth mode. This is because the change of the span of leg lf2 is far from perpendicular to the line connecting the vibration source and leg–ground contact position (figure 9). In addition, there is still no obvious shift of the resonance frequencies.

By sensing different vibration intensities through varying the leg span and angle, the spider may be able to determine the direction of the vibration source relative to the leg orientation, which may be used as a directionality cue for the spider to localize the vibration source. Essentially, whether a change in leg span or angle makes a difference on the FRFs comes down to the leg–ground contact point distance from the vibration source (figure 9). Any change of the span/angle of one leg will only make a significant difference if the distance to the vibration source is altered. In terms of what this could mean biologically, spiders in theory could adjust position of single legs to determine if changes are in line with the vibration source (where in line would result in higher difference in FRF). The magnitude of this effect will also be changed by the degree of attenuation and wave speed of the vibration through the substrate, as this governs how much the vibration alters with distance from the source [5] (in terms of amplitude and phase respectively). The effect of the wave speed is therefore investigated next.

#### 4.2.5. Influence of wave speed

**Figure 11.**
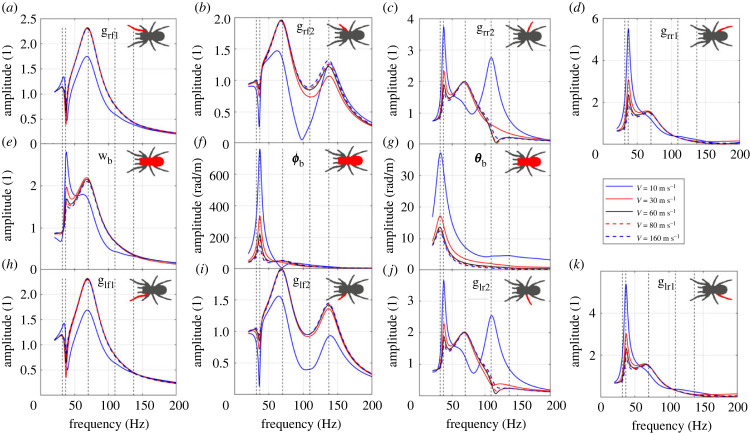
The influence of wave speed on the FRFs to (*a*) leg rf1 bounce; (*b*) leg rf2 bounce; (*c*) leg rr2 bounce; (*d*) leg rr1 bounce; (*e*) body bounce; (*f*) body pitch; (*g*) body roll; (*h*) leg lf1 bounce; (*i*) leg lf2 bounce; (*j*) leg lr2 bounce; (*k*) leg lr1 bounce. The vertical dashed lines represent the natural frequencies of the original system.

The wave speed is mainly dependent on the material properties of the substrate, and sometimes also on the loading [20]. The influence of the wave speed on the FRFs is shown in figure 11. When the speed is greater than 60 m s^−1^, the FRFs do not show significant difference with the increasing speed. This is because higher wave speed results in longer wavelength, and when the size of the spider is significantly smaller than the wavelength, the inputs are close to be the same if the decay rate is zero. So the FRFs will converge with the increase of the wave speed. For example, when the speed is 60 m s^−1^, for a wave frequency between 20 and 200 Hz, the wavelength is between 0.3 and 3 m, which is much larger than the spider size. For speeds less than 60 m s^−1^ at this frequency range, the wavelength is comparable to the spider size, so there are significant differences between the leg inputs, and the FRFs vary significantly with the changing speed.

The wave speed does not change the dynamic properties of the spider, so it influences the FRFs globally. Thus, it can be expected that the wave speed will not affect how different parameters (i.e. body mass, leg stiffness, damping, leg span and angle) influence the FRFs. However, it will affect what vibration inputs go into each leg, thus it will affect the mechanism of localization [5].

## 5. Synthesis and conclusion

Here we investigated the vibration transmission through the spider body, which is an important factor in vibration sensing, yet has been understudied compared to vibration propagation through the environment (e.g. orb webs). To investigate the dynamic properties and vibration transmission through the body, a modal test and lumped parameter modelling for the spider (parametric study) were carried out, respectively.

For the modal test, modelling and experimental data were compared for calibration and validation, which showed five modes of the spider in the frequency range of 20–200 Hz, but no significant vibration above 200 Hz. For broadband prey-generated vibrations less than 1000 Hz, spider body resonance is therefore unlikely to play a role in amplifying biologically relevant vibrations greater than 200 Hz.

For the parametric study, the results showed that the vibration transmissions from different leg inputs caused substantial differences in overall spider dynamics, including what is sensed at each leg. This interaction within the whole spider system means that whole body spider dynamics need to be taken into account when understanding vibration sensing at any one part or leg. This supports the idea that the changing morphology, whether geometry or properties, will influence vibration sensing inputs into the nervous system, as a type of morphological computation [8]. In particular, our results suggest that the changes of biomechanical parameters in front and back legs can influence the leg responses more heavily compared to the middle legs. This effect could be used to help spiders localize vibration sources. More generally, it also means that to understand how vibration sources may be localized in any animal sensing vibrations through their legs, we need to understand how vibration dynamics of the whole system interact with the vibration surface. This applies to vast numbers of arthropods from spiders, to crabs, to insects.

The effects of biomechanical parameters investigated in the parametric study are influenced by multiple interacting biological processes within a single individual, from hunger and dehydration, through developmental stage (moult, growth, reproduction) [34–36,49]. Therefore, we expect physical constraints to act on vibration sensing, as these biological processes will affect mass, inertia, stiffness and damping of biological materials outside of spiders' control. However, some parameters in theory can be actively controlled by the spider, including changing leg stiffness and damping, leg span and angle via muscle action and/or hydrostatic pressure to alter the vibration intensity and spectrum detected [31,32]. Of these, we propose that changing posture is the most likely mechanism used to make small changes to spider biomechanics to influence vibration sensing over small timescales, as it will have influences on leg geometry and properties, as well as mass distribution. Outside of intra-individual variation, it is also possible that different individuals within a population or species have different strategies for how they mitigate the effect of physical constraints on vibration sensing.

Future research should focus on more advanced dynamical modelling of the spider and more complicated excitations. We would also encourage studies that collect empirical data on how spiders are influenced by and respond to biological processes that influence their biomechanics, whether development, malnutrition or leg loss and how this varies between species in different environments.

## Data Availability

The datasets and code supporting this article have been provided as part of the electronic supplementary material [52].
